# Antenatal environmental exposure to indoor air pollution and environmental tobacco smoke: association with birth outcomes in an African birth cohort

**DOI:** 10.1136/bmjresp-2025-003721

**Published:** 2026-06-17

**Authors:** Aneesa Vanker, Kirsty Brittain, Whitney Barnett, Heather J Zar

**Affiliations:** 1 Department of Paediatrics and Child Health and SAMRC Unit on Child and Adolescent Health, University of Cape Town, Red Cross War Memorial Children’s Hospital, Rondebosch, South Africa; 2 Department of Public Health, School of Global Health, Meharry Medical College, Meharry Medical College, Nashville, Tennessee, USA

**Keywords:** Paediatric Physician, Tobacco and the lung

## Abstract

Indoor air pollution (IAP) and environmental tobacco smoke exposure (ETS) are important antenatal environmental exposures that may adversely impact birth outcomes.

**Objectives:**

To investigate the impact of IAP and ETS during pregnancy on birth outcomes including respiratory distress in an African birth cohort, the Drakenstein Child Health Study.

**Setting:**

Prospective cohort study conducted in a periurban setting in sub-Saharan Africa within a large birth cohort.

**Participants:**

Consenting pregnant women (n=1137) enrolled between 20 and 28 weeks’ gestation, with 1143 live births included in the analysis. Participants were recruited antenatally, with inclusion based on residency in the study area and availability of exposure and outcome data.

**Primary and secondary outcome measures:**

Primary exposures included antenatal IAP (particulate matter (PM10), carbon monoxide (CO), nitrogen dioxide, sulphur dioxide, volatile organic compounds) and ETS (maternal urine cotinine). Outcomes included birth anthropometry, prematurity, low birth weight and neonatal respiratory distress.

**Results:**

Among 1143 live births, 17% were premature, 15% had low birth weight and 7% had respiratory distress. Maternal active smoking was associated with reduced weight-for-age z-scores (WfAz; β −0.54; p<0.001). Babies exposed to PM_10_ above ambient standards were 1.88 times more likely to experience respiratory distress compared with those with exposure below ambient standards (p=0.058). Similarly, exposure to CO above ambient standards was associated with a trend towards a doubled odds of respiratory distress in babies at birth (OR: 2.19 (0.97 to 4.98); p=0.061).

**Conclusions:**

Antenatal exposure to IAP and ETS is associated with adverse birth outcomes, including impaired growth and increased risk of respiratory distress. These findings support the need for targeted public health interventions to reduce exposure during pregnancy and highlight the importance of improving air quality to mitigate long-term respiratory health risks.

**WHAT IS ALREADY KNOWN ON THIS TOPIC**

- Air pollution and tobacco smoke exposure have lifelong health impacts starting in early life, but measured exposure data, especially from low and middle-income country African settings, are scarce and their effects on birth outcomes remain poorly described.

**WHAT THIS STUDY ADDS**

- This study provides measured epidemiological data on household air pollution and tobacco smoke exposure in a peri-urban African context and demonstrates their association with key birth outcomes, including respiratory distress, an area with previously limited evidence.

**HOW THIS STUDY MIGHT AFFECT RESEARCH, PRACTICE OR POLICY**

- The findings highlight the urgent need for universal access to clean energy and support targeted antenatal public health interventions to reduce exposure, with the goal of improving long-term health outcomes.

## Introduction

 Air pollution exposure is well recognised as a global health emergency,^1^ and indoor or household air pollution is especially problematic in many African countries as well as other low and middle-income countries (LMIC) globally, where access to clean fuels for household energy is limited.^2^ Furthermore, the burden of tobacco smoke exposure in pregnant women remains a public health concern given the potential adverse impact on the health of both the mother and fetus and is often under-reported.^3^

The antenatal period is a vulnerable time with the developing fetus at risk from noxious environmental exposures. Increasing evidence describes the susceptibility of pregnant women to air pollution with transplacental spread of pollutants and widespread distribution of black carbon and other pollutants in the organs of the unborn fetus.^4 5^ This is particularly important given that in utero exposure has been shown to impact not only birth outcomes but also longitudinal health trajectories.^6 7^ While the impact of tobacco smoke exposure on foetal development and birth outcomes has been well described,^8 9^ the impact of both environmental tobacco smoke (ETS) and indoor air pollution (IAP) exposure on birth outcomes remains limited, particularly in LMIC and African settings. Recent studies from Africa have focused on particulate matter (PM_2.5_) or carbon monoxide (CO) exposure and their impact on birth outcomes, in particular, birth weight, prematurity and perinatal mortality.^10^,^13^ However, few studies have compared the impact of multiple individual household pollutants, nor included explorations of the effect of tobacco smoking and exposure on birth outcomes.^14^ Many pollutants contribute to IAP including particulate matter (PM), CO and volatile organic compounds (VOC), benzene and toluene. Tobacco smoke is an important contributor to IAP, especially in areas where smoking prevalence is high and household ventilation poor.^15 16^ As epidemiological data on IAP and ETS in Africa are limited with many studies still relying on self-reported exposure,^17^ comprehensively assessing multiple types and sources of IAP, including ETS, is important to identify key drivers of birth outcomes to appropriately inform public health interventions.

Therefore, we aimed to investigate the impact of multiple sources of IAP and ETS on birth outcomes in a South African birth cohort study, the Drakenstein Child Health Study (DCHS).^18^

## Methods

A prospective study of antenatal exposure to IAP and ETS in the DCHS was done. Pregnant women in a peri-urban area were enrolled at 20–28 weeks’ gestation at two primary healthcare clinics, Newman (serving a predominantly mixed-ancestry population) and Mbekweni (predominantly black African population), using convenience sampling from 1 March 2012 to 31 March 2015, to ensure enrolment across different time periods and seasons. Written informed consent, in the participants’ home language (English, Afrikaans or isiXhosa), was obtained at enrolment. Antenatal care was provided free of charge at these public sector clinics; all births or any intercurrent hospitalisations occurred at the single central public hospital, Paarl Hospital. Participant demographics were collected at enrolment, objective measures of IAP and ETS were collected at 28–32 weeks’ gestation, and birth data along with a second measure of ETS were collected at delivery or abstracted from hospital folders. Gestational age (GA) at delivery (in weeks) was calculated from an antenatal ultrasound in the second trimester; if this was not available, then fundal height at enrolment or maternal recall of last menstrual period was used.

### Assessing environmental exposures

#### Measuring IAP exposure

An antenatal home visit, conducted by trained fieldworkers, between 28 and 32 weeks’ gestation was done to assess the home environment and to measure IAP using devices placed within the home. Data gathered included access to electricity, types of alternate fuels used for household energy, household size and position of dwelling in relation to passing traffic.^16^ Dwellings were categorised using six factors: type of home (formal vs informal), primary building material (brick or cement vs other materials), water supply (piped into dwelling or yard), toilet facilities (non-communal flush), kitchen type (separate room in house) and ventilation in the kitchen area (pipe or duct to exterior).^16^ A home lacking two or more of these dimensions was categorised as a poor structure.^19 20^

To assess IAP, the most common pollutants and by-products of combustion were measured. On consultation with air quality specialists (SGS^R^ environmental), personal samplers were used based on available, validated equipment. Devices were placed in participants’ homes, with all measurements done in the communal/main living room, away from windows and doors, approximately 1.5 m from the ground, as previously described.^16^ Devices were left in the home for 24 hours to 2 weeks depending on the measurement.^16^ Particulate matter (PM_10_) was measured using a personal air sampling pump [SKC AirChek 52^R^] and CO with an Altair^R^ carbon monoxide single gas detection unit, left in homes for 24 hours.^16^ Diffusion tubes placed in homes for 2 weeks measured nitrogen dioxide (NO_2_)/sulphur dioxide (SO_2_; Radiello^R^ adsorbent filters in polyethylene diffusive body) and VOC, benzene and toluene (Markes^R^ thermal desorption tubes).^16^ An average concentration based on the 2-week duration in the home was obtained for NO_2_/SO_2_ and VOC; 24-hour averages were obtained for PM_10_. CO data were downloaded to a computer and the frequency of exceedance above the hourly ambient standard was calculated.^16^ The South African National Ambient Air Quality Standards^21^ were used to define expected exposure levels for each pollutant based on an averaging period of 1 year for each measure; PM_10_: 40 µg/m^3^, NO_2_: 40 µg/m^3^, SO_2_: 50 µg/m^3^, benzene: 5 µg/m^3^, toluene: 240 µg/m^3^; and based on a 1-hour average for CO: >30 mg/m^3^ (not more than 88 hours).^21^

#### Measuring ETS exposure

As a metabolite of nicotine and a robust proxy for maternal smoking or exposure to tobacco smoke, maternal urine cotinine was measured at the second antenatal visit (28–32 weeks’ gestation) and again at birth; the highest result was used to assign smoking status.^22^ Urine cotinine tests were performed using IMMULITE^R^ 1000 Nicotine Metabolite Kit (Siemens Medical Solutions Diagnostics^R^, Glyn Rhonwy, United Kingdom). This provided a quantitative test using a competitive chemiluminescent immunoassay, which contained solid-phase beads coated with polyclonal rabbit anticotinine antibody. The test had a calibration range of 10–500 μg/mL, with an analytical sensitivity of 2 ng/mL.^23^ Urine cotinine levels were classified as <10 ng/mL (non-smoker), 10–499 ng/mL (passive smoker/exposed), or ≥500 ng/mL (active smoker) according to the manufacturer’s directions and the testing laboratory’s limits.^22^

#### Birth outcomes

All births were attended by a member of the study team. Birth outcomes included birth weight, prematurity (<37 weeks GA) and presence of respiratory distress. birth weight and length were measured in the labour ward by trained clinical staff as part of routine care and obtained from hospital records. Infant weight was measured to the nearest 10 g and length to the nearest completed 0.5 cm according to standard operating procedures.^24^ GA at birth was estimated based on a second trimester antenatal ultrasound where available (68%); if ultrasound was unavailable, GA was estimated using fundal height (28%) or maternal recall of the last menstrual period (4%).^24^ In a subgroup of participants, labour ward anthropometric measurements were compared with study staff measurements and no significant differences were observed, supporting measurement reliability.

Weight-for-age z-scores (WfAz) at birth were calculated using the WHO standards for full-term infants and the Fenton growth charts for preterm infants.^25 26^ Respiratory distress was defined as a documented respiratory problem (grunting, fast breathing, recession, cyanosis or apnoea) or receipt of supplemental oxygen or respiratory support at birth.

### Statistical analysis

Data were analysed using STATA V.14.2 (Stata Corporation, College Station, Texas). In descriptive analyses, maternal, household and birth characteristics were summarised across study site, and IAP and maternal urine cotinine were summarised using the ambient standards and urine cotinine thresholds described above. The effects of IAP and maternal urine cotinine on WfAz at birth were explored in linear regression models, and the effects on preterm birth and respiratory distress after birth were explored in logistic regression models. Throughout, a two-sided p value of <0.05 was considered to be statistically significant, and results are presented with 95% CIs. Confounders were selected a priori based on directed acyclic graphs (online supplemental figures S1 and S2) and included maternal education and household income. In sensitivity analyses, models were additionally adjusted for maternal age at enrolment and maternal HIV infection (online supplemental table S1).

Sensitivity analyses were conducted to assess the robustness of the findings. These included models stratified by study site (online supplemental table S2) and analyses examining associations between maternal smoking and birth outcomes using alternative cotinine-based smoking classifications (online supplemental table S3).

### Patient and public participation

While patients or the public were not directly involved in the design or conduct of the research, local community members played an important role in its implementation. Fieldworkers were recruited from within the study communities to support data collection, which enhanced trust and cultural relevance. In addition, community engagement activities were carried out to assess the acceptability of home visits and the placement of measuring equipment in households.

## Results

**Figure 1:**
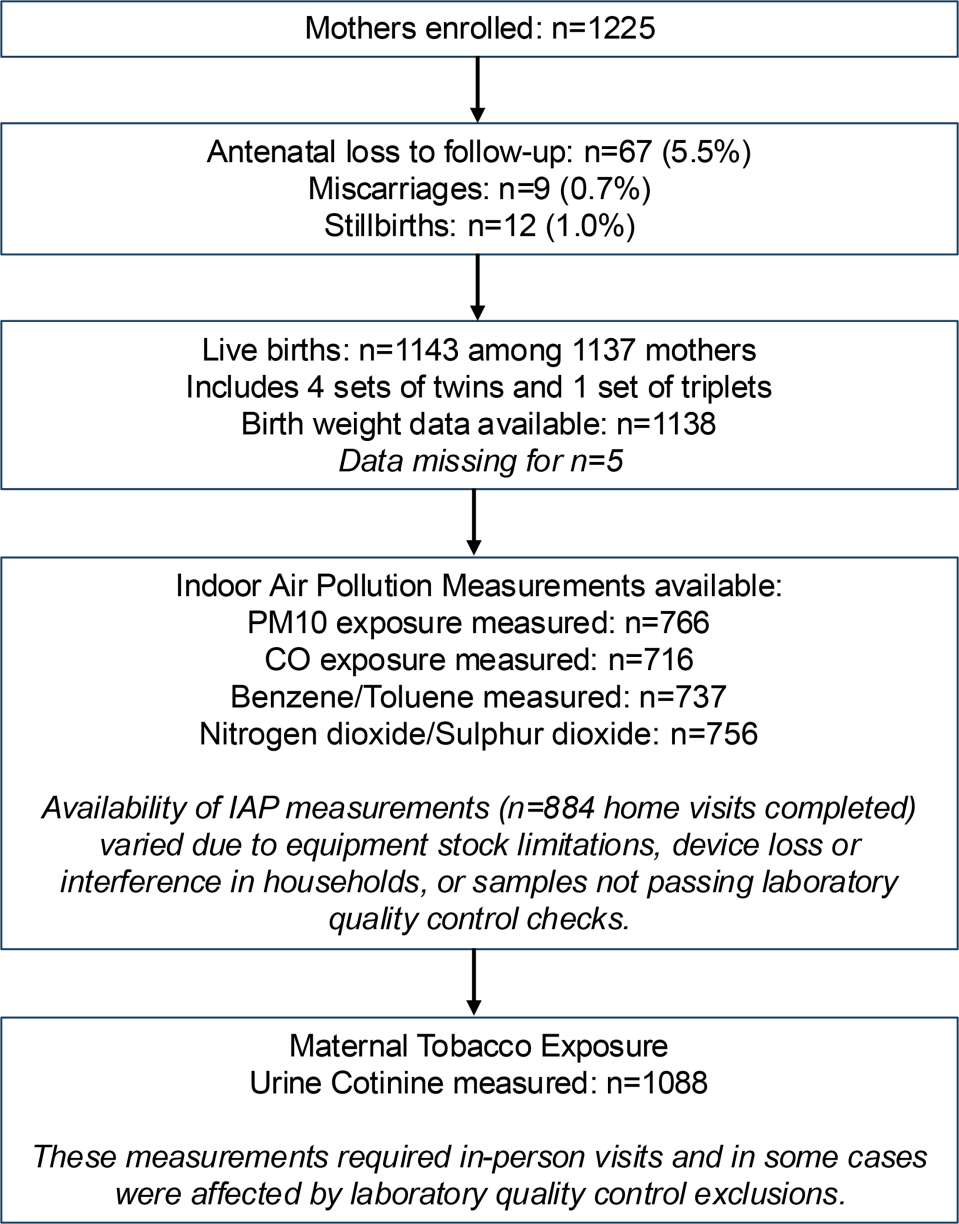
Study flow diagram. CO, Carbon monoxide; IAP, indoor air pollution; PM10, particulate matter.

Of 1225 mothers enrolled, 67 (5.5%) were lost to follow-up prior to delivery; 9 (0.7%) experienced miscarriages; and 12 (1.0%) experienced stillbirths, resulting in 1143 live births among 1137 mothers (figure 1). Median maternal age was 25.8 (IQR 22.0–30.8) years and 392 (35%) were primigravid, with 244 (21%) HIV-infected. Almost all HIV-infected women (97%) were taking antiretroviral therapy. Levels of education were low, with 39% of mothers having completed secondary or any tertiary education (n=445); 51% of households had at least one parent employed (n=578); and most households had an average monthly income of less than ZAR 5000 (US$260). Gestational complications were diagnosed in a minority of women, including hypertension in 52 women (5%), pre-eclampsia in 29 (3%) and asthma in 17 (2%; table 1).

**Table 1:** Maternal and household characteristics by study site

Characteristics	Total	Mbekweni	Newman
Number of mothers	1137	628	509
Median (IQR) age at enrolment	25.8 (22.0, 30.8)	26.7 (22.3, 31.6)	24.7 (21.4, 29.2)
Married/cohabiting	458 (40%)	237 (38%)	221 (43%)
Primigravid	392 (35%)	200 (32%)	192 (38%)
Education			
Primary/some secondary	692 (61%)	389 (62%)	303 (60%)
Completed secondary/any tertiary	445 (39%)	239 (38%)	206 (40%)
Parent employed	578 (51%)	300 (48%)	278 (55%)
Average household income (ZAR)			
<R1000/month	385 (34%)	233 (37%)	152 (30%)
R1000–R5000/month	592 (52%)	327 (52%)	265 (52%)
>R5000/month	159 (14%)	68 (11%)	91 (18%)
Household density—median (IQR)			
Household size	4 (3, 6)	4 (3, 6)	5 (4, 7)
Persons per room (n=834)	2 (1, 2)	2 (1, 3)	1 (1, 2)
Persons per sleeping room (n=830)	3 (2, 4)	3 (2, 4)	3 (2, 5)
Household dimensions			
Type of home: formal (n=836)	553 (66%)	270 (61%)	283 (72%)
Material: brick/cement (n=832)	548 (66%)	270 (61%)	278 (71%)
Water supply: piped (n=825)	721 (87%)	378 (86%)	343 (89%)
Toilet: non-communal flush (n=832)	597 (72%)	327 (74%)	270 (69%)
Kitchen type: separate room (n=821)	359 (44%)	87 (20%)	272 (72%)
Kitchen ventilation: pipe/duct (n=821)	37 (5%)	37 (8%)	0 (0%)
Household dimension categories (n=836)			
≤2 dimensions	278 (33%)	172 (39%)	106 (27%)
>2 dimensions	558 (67%)	271 (61%)	287 (73%)
Electricity in the home (n=834)	773 (93%)	393 (89%)	380 (97%)
Fossil fuel (coal, wood, paraffin, gas) used			
Cooking (n=816)	175 (21%)	138 (32%)	37 (10%)
Heating (n=524)	148 (28%)	140 (35%)	8 (6%)
Type of stove (n=816)			
Electric	734 (90%)	378 (86%)	356 (94%)
Paraffin	127 (16%)	123 (28%)	4 (1%)
Gas	55 (7%)	23 (5%)	32 (8%)
Wood	3 (0.4%)	2 (0.5%)	1 (0.3%)
Coal	2 (0.3%)	2 (0.5%)	0 (0%)
Distance of home from continuously passing trucks (n=833)			
Passing trucks (n=833)			
<50 m	380 (46%)	287 (65%)	93 (24%)
50–100 m	129 (15%)	94 (21%)	35 (9%)
100–200 m	91 (11%)	56 (13%)	35 (9%)
200–500 m	95 (11%)	3 (0.7%)	92 (23%)
>500 m	138 (17%)	1 (0.2%)	137 (35%)
HIV-infected	244 (21%)	228 (36%)	16 (3%)
On antiretroviral therapy (n=244)	237 (97%)	221 (97%)	16 (100%)
Maternal health complications diagnosed during pregnancy			
Diabetes	14 (1%)	7 (1%)	7 (1%)
Preeclampsia	29 (3%)	15 (2%)	14 (3%)
Hypertension	52 (5%)	30 (5%)	22 (4%)
Asthma	17 (2%)	7 (1%)	10 (2%)
Anaemia	250 (22%)	161 (26%)	89 (17%)

Data presented as n (%) of mothers or median with interquartile range (IQR).

ZAR, South African rand.

### Home environment, IAP and ETS exposures

A total of 884 home visits were conducted antenatally. From the home visits conducted, the majority 66% (n=553) were formal structures, but 278 (33%) had two or fewer of the basic household dimensions. More Mbekweni homes (39%) had two or fewer basic household dimensions compared with Newman (27%). Although most homes had access to electricity (93%), fossil fuels were still used for cooking (21%) or heating (28%), with more Mbekweni than Newman homes using fossil fuels for cooking or heating (table 1).

Of the 884 home visits completed antenatally, IAP data were available for PM_10_ levels in a total of 764 homes, benzene in 735, CO in 714, and NO_2_ and SO_2_ in 756 (see figure 1). Incomplete IAP data primarily reflected equipment stock limitations, device loss or interference in households or samples not passing laboratory quality control checks. Almost half of homes had measured PM_10_ (45%) and benzene (45%) levels above acceptable ambient standards with no significant differences between sites. For CO levels, 11% of homes were above acceptable ambient standards. However, for NO_2_ and SO_2_, the proportion of homes with levels above ambient standards was very low (3% and 0.5%, respectively), and these pollutants were, therefore, not included in the analyses of the impacts of IAP on birth outcomes (table 2).

**Table 2:** Antenatal indoor air pollution and environmental tobacco smoke (maternal urine cotinine) by study site

Measure	Total	Mbekweni	Newman
Number of mothers	1137	628	509
Particulate matter (PM_10_) g/m^3^ (n=764)	33.4 (12.4, 64.8)	31.9 (12.3, 62.5)	35.7 (12.8, 66.1)
PM_10_ above ambient standards	344 (45%)	174 (43%)	170 (47%)
Nitrogen dioxide (NO_2_) µg/m^3^ (n=756)	7.1 (3.3, 12.7)	6.9 (2.5, 14.6)	7.2 (3.8, 11.3)
NO_2_ above ambient standards	19 (3%)	16 (4%)	3 (0.8%)
Sulphur dioxide (SO_2_) µg/m^3^ (n=756)	0.0 (0.0, 0.3)	0.0 (0.0, 0.3)	0.0 (0.0, 0.2)
SO_2_ above ambient standards	4 (0.5%)	4 (1%)	0 (0%)
Carbon monoxide (CO) mg/m^3^ (n=714)	0(0, 110)	0(0, 65)	0(0, 120)
CO above ambient standards	80 (11%)	39 (10%)	41 (13%)
Benzene µg/m^3^ (n=735)	4.3 (1.8, 11.4)	4.5 (1.5, 17.7)	3.9 (1.8, 8.6)
Benzene above ambient standards	333 (45%)	184 (47%)	149 (43%)
Toluene µg/m^3^ (n=735)	16.8 (7.1, 44.3)	16.0 (5.9, 42.9)	17.4 (8.2, 46.0)
Toluene above ambient standards	67 (9%)	36 (9%)	31 (9%)
Maternal urine cotinine (n=1088)			
Non-smoker	249 (23%)	196 (33%)	53 (11%)
Passive smoker/exposed	486 (45%)	308 (52%)	178 (36%)
Active smoker	353 (32%)	91 (15%)	262 (53%)

Data presented as n (%) of mothers or median with interquartile range (IQR). South African National Ambient Air Quality Standards based on an averaging period of 1 year: PM_10_: 40 µg/m3, NO_2_: 40 µg/m3, SO_2_: 50 µg/m3, benzene: 5 µg/m3, toluene: 240 µg/m3; and based on a 1 hour average for CO: >30 mg/m3 (not more than 88 hours). Maternal urine cotinine as a proxy for environmental tobacco smoke: <10 ng/mL (non-smoker), 10-499 ng/mL (passive smoker/exposed) or ≥500 ng/mL (active smoker).

Overall, 1088 mothers had urine cotinine measurements (493 from Newman and 595 from Mbekweni). Cotinine measurements were obtained during in-person maternal visits, and missing data were primarily due to missed study visits rather than laboratory processing issues. Based on these measurements, maternal active tobacco smoking prevalence was 32% overall, much higher for Newman mothers compared with Mbekweni mothers (53% vs 15%). A further 45% of mothers were classified as passive smokers, with a higher prevalence in Mbekweni mothers compared with Newman mothers (52% vs 36%; table 2).

#### Birth outcomes

There were 1143 live births, 53% male, with median GA 39 (IQR 37, 40) weeks and 17% (189) were preterm (<37 weeks gestation), of which 128 (68%) were late preterm (≥34 weeks gestation). Vaginal deliveries occurred in 906/1136 births (80%). The median birth weight was 3080 (IQR 2710–3420) g, with Mbekweni babies weighing more than Newman babies (median 3150 vs 3000). The median WfAz was −0.25 (IQR −0.93–0.40). Respiratory distress was found in 76/1143 (7%) babies at birth, with no differences across site. Most babies with respiratory distress (58/76; 76%) required hospital admission (table 3).

**Table 3:** Birth outcomes by study site

Measure	Total	Mbekweni	Newman
Number of infants	1143	634	509
Sets of twins	4	4	0
Sets of triplets	1	1	0
Sex—male	586 (51%)	309 (49%)	277 (54%)
Median (IQR) gestation at delivery	39 (37, 40)	39 (38, 40)	39 (37, 40)
Pre-term birth (<37 weeks gestation)	189 (17%)	106 (17%)	83 (16%)
Median (IQR) gestation at delivery among pre-term births (n=189)	35 (33, 36)	35 (33, 36)	35 (32, 36)
Vaginal delivery (n=1136)	906 (80%)	481 (77%)	425 (84%)
Median (IQR) birth weight in grams (n=1138)	3080 (2710, 3420)	3150 (2780, 3450)	3000 (2630, 3350)
Median (IQR) weight-for-age z-score (n=1138)	−0.25 (−0.93, 0.40)	−0.13 (−0.80, 0.49)	−0.43 (−1.09, 0.23)
Low birth weight (<2500 grams; n=1138)	176 (15%)	81 (13%)	95 (19%)
Respiratory problems at birth	76 (7%)	43 (7%)	33 (6%)
Hospitalised with respiratory problems	58 (5%)	31 (5%)	27 (5%)

Data presented as n (%) of infants or median with interquartile range (IQR).

#### Associations between IAP, maternal urine cotinine and birth outcomes

No associations were observed between IAP and WfA z-scores at birth in either unadjusted models or models adjusted for maternal education and household income (table 4). However, high maternal urine cotinine levels (indicating active smoking) were significantly associated with a decrease in WfA z-score after adjustment for maternal education and household income (β coefficient: −0.54; 95% CI −0.71 to −0.37; p<0.001). Neither IAP nor maternal urine cotinine were significantly associated with preterm birth in unadjusted analyses or after adjustment for maternal education and household income. Exposure to PM_10_ or CO above ambient standards was associated with an increased odds of respiratory distress at birth, although these associations did not reach statistical significance. After adjustment for maternal education and household income, babies exposed to PM_10_ above ambient standards were 1.88 times more likely to experience respiratory distress compared with those with exposure below ambient standards (95% CI 0.98 to 3.61; p=0.058). Similarly, exposure to CO above ambient standards more than doubled the odds of respiratory distress in babies at birth (OR: 2.19; 95% CI 0.97 to 4.98; p=0.061). Results were unchanged after additional adjustment for maternal age at enrolment and maternal HIV infection (online supplemental table S1).

**Table 4:** Impact of indoor air pollution above ambient standards and environmental tobacco smoke (maternal urine cotinine) on infant birth outcomes

(*A) Weight-for-age z-score at birth, among 1138 babies with birthweight data*					
	n	**Unadjusted model**		**Adjusted model**	
		Unadjusted β (95% CI)	P value	Adjusted β (95% CI)	P value
Particulate matter (PM_10_)	766	−0.08 (−0.22 to 0.06)	0.270	−0.06 (−0.20 to 0.08)	0.414
Carbon monoxide (CO)	716	−0.07 (−0.30 to 0.16)	0.553	−0.08 (−0.31 to 0.15)	0.491
Benzene	737	−0.05 (−0.19 to 0.10)	0.523	−0.03 (−0.17 to 0.12)	0.715
Toluene	737	−0.13 (−0.38 to 0.12)	0.317	−0.13 (−0.38 to 0.12)	0.323
Maternal urine cotinine (vs non-smoker, n=249)					
Passive smoker/exposed	490	−0.10 [−0.26, 0.05]	0.196	−0.10 [−0.26, 0.05]	0.204
Active smoker	353	−0.55 [−0.72, -0.39]	<0.001	−0.54 [−0.71, -0.37]	<0.001
*(B) Preterm vs full-term birth, among 1143 babies*					
	n	**Unadjusted model**		**Adjusted model**	
		Unadjusted OR (95% CI)	P value	Adjusted OR (95% CI)	P value
PM_10_	767	1.07 (0.70 to 1.64)	0.765	1.05 (0.68 to 1.61)	0.836
CO	716	1.45 (0.76 to 2.76)	0.255	1.41 (0.74 to 2.69)	0.304
Benzene	738	1.18 (0.75 to 1.85)	0.478	1.14 (0.72 to 1.79)	0.578
Toluene	738	1.03 (0.47 to 2.24)	0.939	1.02 (0.47 to 2.22)	0.957
Maternal urine cotinine (vs non-smoker, n=249)					
Passive smoker/exposed	491	1.09 [0.70, 1.68]	0.713	1.03 (0.66, 1.60)	0.905
Active smoker	353	1.33 (0.85, 2.08)	0.216	1.20 (0.75, 1.90)	0.444
*(C) Respiratory distress vs no respiratory distress at birth, among 1143 babies*					
	n	**Unadjusted model**		**Adjusted model**	
		Unadjusted OR (95% CI)	P value	Adjusted OR (95% CI)	P value
PM_10_	767	1.90 (0.99 to 3.63)	0.053	1.88 (0.98 to 3.61)	0.058
CO	716	2.10 (0.93 to 4.73)	0.074	2.19 (0.97 to 4.98)	0.061
Benzene	738	1.61 (0.84 to 3.08)	0.154	1.63 (0.85 to 3.14)	0.143
Toluene	738	1.15 (0.40 to 3.35)	0.793	1.14 (0.39 to 3.31)	0.811
Maternal urine cotinine (vs non-smoker, n=249)					
Passive smoker/exposed	491	0.62 (0.33 to 1.16)	0.135	0.63 (0.34 to 1.19)	0.155
Active smoker	353	1.04 (0.57 to 1.91)	0.892	1.04 (0.55 to 1.95)	0.904

β: β coefficient from linear regression model. Adjusted models: adjusted for maternal education and household income. South African National Ambient Air Quality Standards based on an averaging period of 1 year: PM_10_: 40 µg/m3, benzene: 5 µg/m3, toluene: 240 µg/m3; and based on a 1 hour average for CO: >30 mg/m3 (not more than 88 hours). Maternal urine cotinine as a proxy for environmental tobacco smoke: <10 ng/mL (non-smoker), 10–499 ng/mL (passive smoker/exposed) or ≥500 ng/mL (active smoker).

In sensitivity analyses stratified by study site, associations between environmental exposures and birth outcomes were broadly consistent with those observed in the pooled analyses (online supplemental table S2). Additional sensitivity analyses using alternative categorisations of maternal smoking based on urine cotinine also produced similar findings (online supplemental table S3).

## Discussion

In this study, in a poor peri-urban South African population, we found high levels of maternal urine cotinine, a proxy for ETS, and of exposure to IAP especially to PM_10_ and benzene, with almost half of all households being exposed to levels above ambient standards. Maternal urine cotinine above the threshold indicating active smoking was associated with lower weight-for-age in infants at birth, consistent with prior studies.^22 27 28^ We also found that higher antenatal exposure to PM_10_ and CO trended towards higher odds of respiratory distress.

Systematic reviews have shown associations between household air pollution and birth outcomes, including birth weight, prematurity and neonatal mortality; however, note that the majority of studies rely on self-reported air pollution exposures or modelled IAP exposure and many studies use maternal recall of birth outcomes.^10 29 30^ More recently, studies have assessed the association between particulate matter (PM_2.5_) or CO from household air pollution and the association with birth outcomes and showed a tendency towards prematurity and low birth weight;^11 13^ however, few studies measure both IAP and ETS exposure directly nor do they include respiratory distress as an outcome.^31^ In this study, key pollutants, the result of unclean fuel combustion, were measured in over 700 homes and maternal urine cotinine was quantified in more than 1000 pregnant women, providing robust exposure data. Furthermore, all births were attended by a member of the study team and birth outcomes were clearly documented at the regional hospital by the healthcare providers ensuring accurate outcome assessment. Therefore, the present study adds to this existing literature by examining associations between objectively measured IAP and ETS and rigorously collected birth outcome data.

The association seen between PM_10_ and CO exposure above acceptable ambient standards and respiratory distress at birth is an important finding. While these associations did not reach statistical significance, these findings support the growing body of evidence that antenatal environmental exposures drive in-utero inflammatory and chemotaxic process significantly impacting not only early-life outcomes but also potentially life-long lung health.^32^,^34^ This emphasises the urgent need to reduce antenatal air pollution exposure and to prioritise public health interventions that may mitigate these risks.

The sources of CO exposure may be both from fuel combustion and a by-product of cigarette smoke.^35 36^ Although we did not find an association between measured household CO levels and maternal smoking, this may be the result of multiple household sources of CO. The impact of cigarette smoking on birth weight is still important with potential far-reaching health consequences, including impaired growth and long-term lung health.^37^ We have already shown that antenatal IAP and ETS exposures impact lung function^38^ and are associated with lower respiratory tract infections in infancy.^39^ The findings from this study further highlight that these antenatal exposures are an important determinant of birth outcomes.

Understanding the social determinants of health is complex^40^ and while this was not the primary aim of this study, we found several notable differences in antenatal exposures between the two sites with likely consequences for early-life child health. The distinct sociodemographic and environmental profiles of these two communities provide important context for interpreting exposure patterns in this cohort. There was a higher reliance on alternate fossil fuel sources in the Mbekweni community, potentially a reflection of the slightly lower socioeconomic status of this community. Additionally, there were higher maternal urine cotinine levels in the Newman community, likely due to greater social acceptance of women smoking in this community.^22^ Interestingly, despite these differences, measured IAP exposures were similar across both sites.

Although this study was limited to a single antenatal IAP measurement in the last trimester of pregnancy, the findings remain important given the limited African data on directly measured air pollution exposures.^29^ Future studies should consider measuring IAP at different timepoints throughout pregnancy, and even preconception to improve understanding of exposure timing and outcomes. Furthermore, IAP and ETS have been shown to be associated with stillbirth and perinatal mortality; in this study, the small numbers of miscarriages and still births precluded this analysis. Another important limitation is that PM_10_ and not PM_2.5_ was measured, due to the availability of personal sampling equipment at the time. While PM_2.5_ is recognised to penetrate deeper into the respiratory system and systemic circulation, we showed high numbers of households with levels above ambient standards for PM_10_, which is likely to also reflect exposure to PM_2.5_.^11^ The associations (though not reaching statistical significance) seen with PM_10_ exposure are still important and should be considered in strategies to reduce air pollution exposures. Additionally, we were limited by the types of pollutants that could be measured. Formaldehyde, released from household cleaning products and building materials, is a major IAP that has also been implicated in adverse birth outcomes and longer term health.^41^ However, this was not included in the panel of VOCs measured. Other important pollutants must be included in future studies. Furthermore, future studies with larger samples and more complete exposure measurements should examine the potential joint or interactive effects of IAP and ETS during pregnancy, as these exposures frequently co-occur in many settings. The small numbers of children born premature or with respiratory distress limit the power to detect differences; however, this reflects good antenatal care and follow-up in a public health setting. In addition, although GA was primarily estimated using second trimester ultrasound, approximately 28% of participants had GA estimated using fundal height, which may introduce some measurement imprecision. Finally, urine cotinine measurements were classified according to the manufacturer’s directions, but we recognise the potential for misclassification of smoking status, especially in the ‘passive smoking’ category.

The strengths of this study include (1) the large number of homes visited to obtain objective IAP measurements antenatally; (2) the recruitment period over 3 years allowing for measurements to be collected across seasons and (3) conducting home visits in participant homes removed bias from self-reporting exposures. In addition, high cohort retention through pregnancy and careful phenotyping of infants at birth are key strengths.

## Conclusion

High exposure to IAP and ETS in this study highlights the need for improvement in environmental conditions and greater use of clean fuels in LMICs. The association between maternal urine cotinine and lower weight-for-age at birth underscores the urgent need for effective programmes to address maternal smoking preconception and during pregnancy and to strengthen education about the dangers of IAP exposure to the developing infant. Future work should address potential long-term implications of early-life exposures on developmental trajectories of health.

## Supplementary material

10.1136/bmjresp-2025-003721 online supplemental file 1

## Data Availability

Data are available upon reasonable request.
