# Supplementary material for: Antenatal environmental exposure to indoor air pollution and environmental tobacco smoke: association with birth outcomes in an African birth cohort
Source: BMJ Open Respir Res. 2026 Jun 17;13(1):e003721. doi: 10.1136/bmjresp-2025-003721 (PMC13289235; doi:10.1136/bmjresp-2025-003721)
Supplement: online supplemental file 1 [file bmjresp-13-1-s001.docx]

**Supplementary Materials**

Antenatal environmental exposure to indoor air pollution and environmental tobacco smoke: association with birth outcomes in an African birth cohort.

Aneesa Vanker, Kirsty Brittain, Whitney Barnett, Heather J. Zar

**Supplementary Figure S1** Directed acyclic graph of hypothesised relationships between indoor air pollution and birth outcomes

**Supplementary Figure S2** Directed acyclic graph of hypothesised relationships between maternal tobacco smoking and birth outcomes

**Supplementary Table S1** Sensitivity analyses of the impact of indoor air pollution above ambient standards and environmental tobacco smoke (maternal urine cotinine) on infant birth outcomes, with additional adjustment for maternal age at enrolment and maternal HIV infection

**Supplementary Table S2** Sensitivity analyses examining associations between indoor air pollution above ambient standards and environmental tobacco smoke (maternal urine cotinine) on infant birth outcomes, stratified by study site

**Supplementary Table S3** Sensitivity analyses examining associations between environmental tobacco smoke (maternal urine cotinine, using alternative cotinine classifications) and birth outcomes

**Supplementary Figure S1** Directed acyclic graph of hypothesised relationships between indoor air pollution and birth outcomes


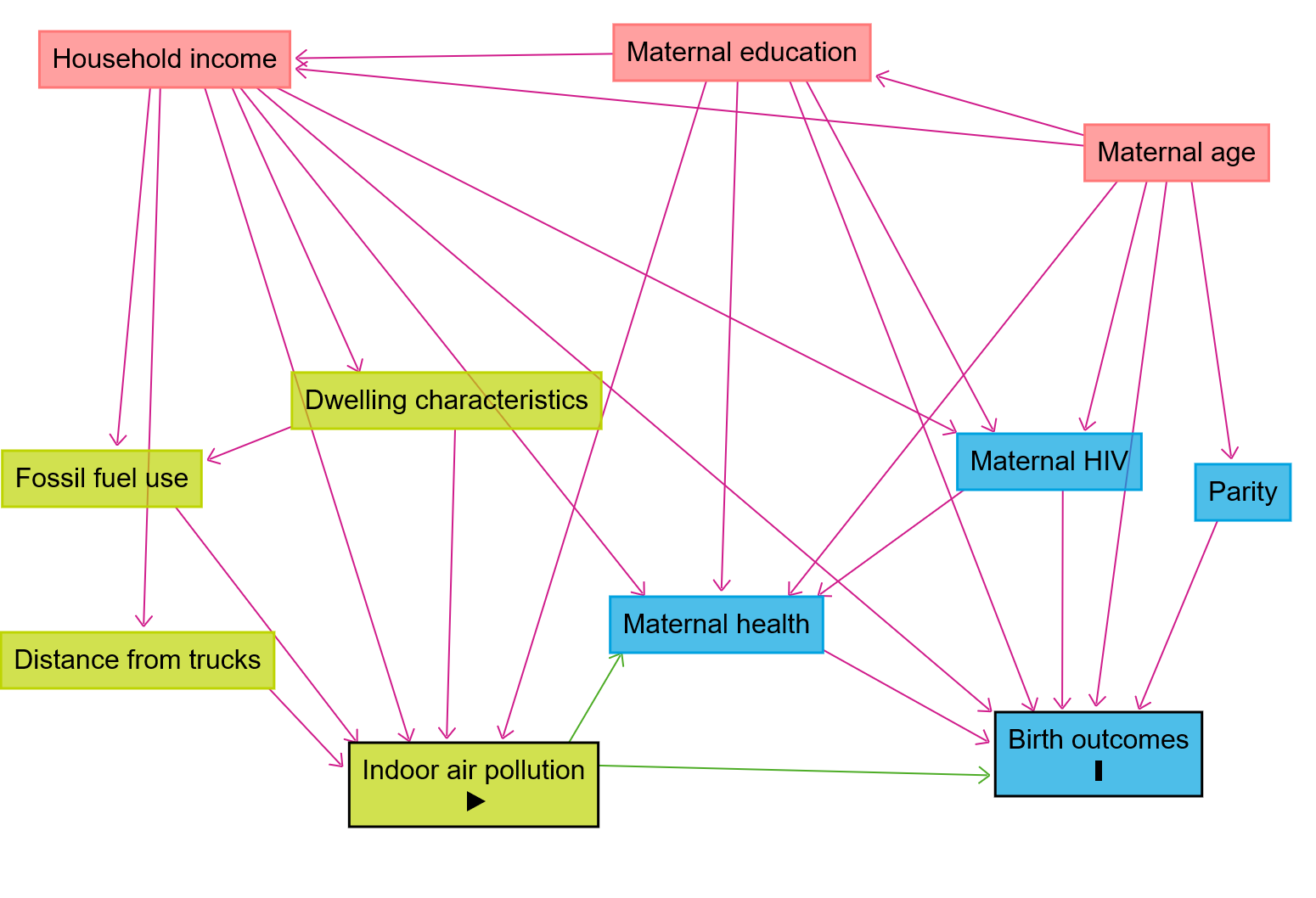


Footnote: Directed Acyclic Graph (DAG) constructed in [www.dagitty.net](http://www.dagitty.net) to examine for possible confounding in the relationship between indoor air pollution and birth outcomes. Minimal sufficient adjustment sets for estimating the total effect of indoor air pollution on birth outcomes include maternal education and household income.


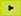
 exposure


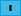
 outcome


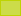
 ancestor of exposure


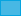
 ancestor of outcome


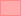
 ancestor of exposure and outcome


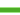
 causal path


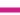
 biasing path

**Supplementary Figure S2** Directed acyclic graph of hypothesised relationships between environmental tobacco smoke (maternal urine cotinine) and birth outcomes


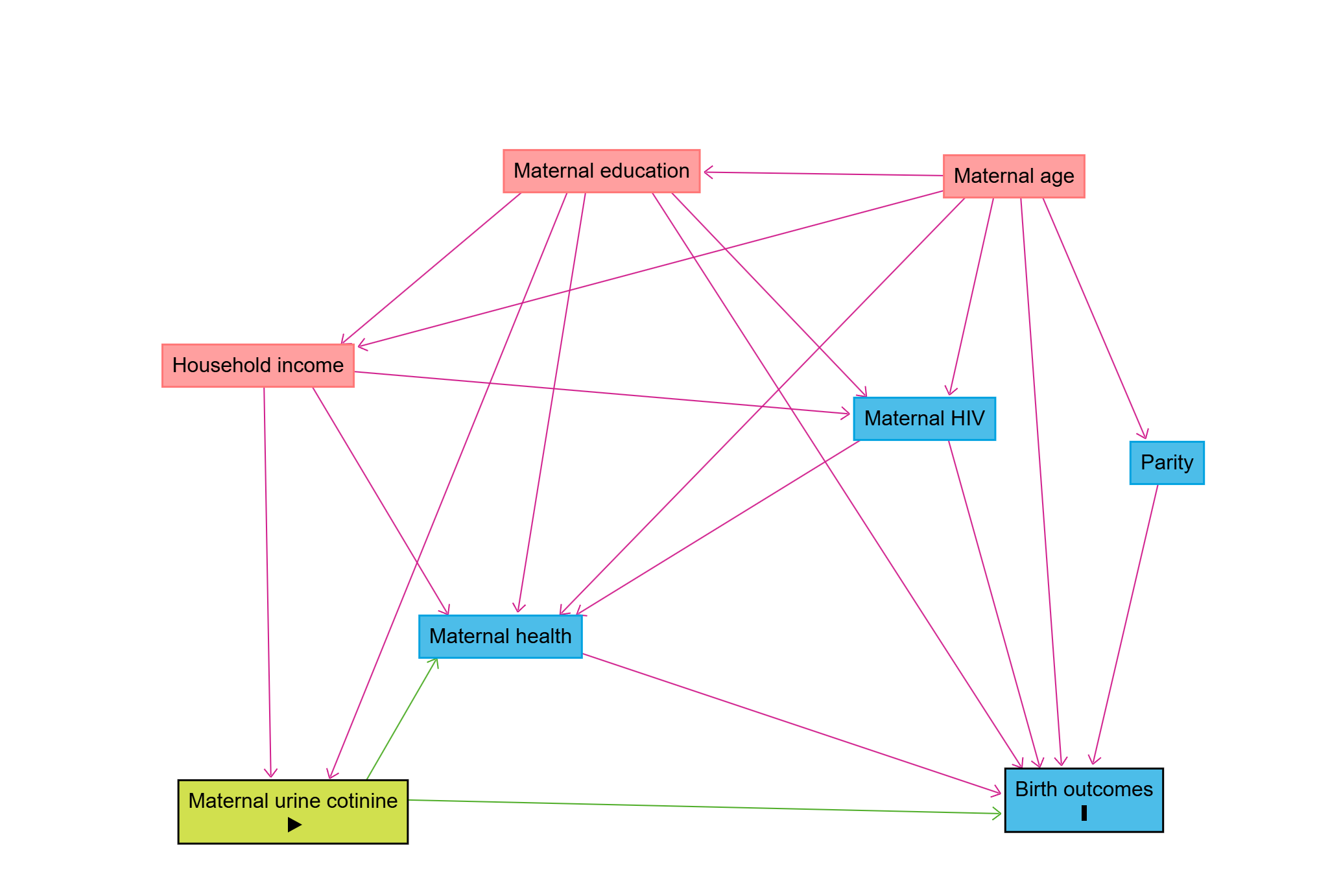


Footnote: Directed Acyclic Graph (DAG) constructed in [www.dagitty.net](http://www.dagitty.net) to examine for possible confounding in the relationship between maternal tobacco smoking and birth outcomes. Minimal sufficient adjustment sets for estimating the total effect of maternal urine cotinine on birth outcomes include maternal education and household income.


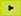
 exposure


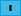
 outcome


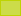
 ancestor of exposure


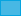
 ancestor of outcome


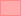
 ancestor of exposure and outcome


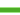
 causal path


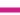
 biasing path

| **Supplementary Table S1** Sensitivity analyses of the impact of indoor air pollution above ambient standards and environmental tobacco smoke (maternal urine cotinine) on infant birth outcomes, with additional adjustment for maternal age at enrolment and maternal HIV infection | | | | | |
| --- | --- | --- | --- | --- | --- |
| ***(A) Weight-for-age z-score at birth, among 1138 babies with birthweight data*** | | | | | |
|  | n | **Unadjusted models** | | **Adjusted model** | |
|  |  | Unadjusted β [95% CI] | P-value | Adjusted β [95% CI] | P-value |
| Particulate matter (PM_10_) | 766 | -0.08 [-0.22, 0.06] | 0.270 | -0.06 [-0.21, 0.08] | 0.393 |
| Carbon monoxide (CO) | 716 | -0.07 [-0.30, 0.16] | 0.553 | -0.08 [-0.32, 0.15] | 0.487 |
| Benzene | 737 | -0.05 [-0.19, 0.10] | 0.523 | -0.03 [-0.18, 0.12] | 0.689 |
| Toluene | 737 | -0.13 [-0.38, 0.12] | 0.317 | -0.13 [-0.38, 0.12] | 0.310 |
| Maternal urine cotinine (vs non-smoker, n=249)  Passive smoker/exposed  Active smoker | 490  353 | -0.10 [-0.26, 0.05]  -0.55 [-0.72, -0.39] | 0.196  <0.001 | -0.10 [-0.25, 0.06]  -0.53 [-0.70, -0.36] | 0.231  <0.001 |
| ***(B) Preterm versus full-term birth, among 1143 babies*** | | | | | |
|  | n | **Unadjusted models** | | **Adjusted model** | |
|  |  | Unadjusted OR [95% CI] | P-value | Adjusted OR [95% CI] | P-value |
| Particulate matter (PM_10_) | 767 | 1.07 [0.70, 1.64] | 0.765 | 1.06 [0.69, 1.63] | 0.782 |
| Carbon monoxide (CO) | 716 | 1.45 [0.76, 2.76] | 0.255 | 1.39 [0.73, 2.67] | 0.317 |
| Benzene | 738 | 1.18 [0.75, 1.85] | 0.478 | 1.14 [0.72, 1.79] | 0.576 |
| Toluene | 738 | 1.03 [0.47, 2.24] | 0.939 | 1.03 [0.47, 2.23] | 0.946 |
| Maternal urine cotinine (vs non-smoker, n=249)  Passive smoker/exposed  Active smoker | 491  353 | 1.09 [0.70, 1.68]  1.33 [0.85, 2.08] | 0.713  0.216 | 1.02 [0.66, 1.59]  1.20 [0.75, 1.91] | 0.923  0.446 |
| ***(C) Respiratory distress versus no respiratory distress at birth, among 1143 babies*** | | | | | |
|  | n | **Unadjusted models** | | **Adjusted model** | |
|  |  | Unadjusted OR [95% CI] | P-value | Adjusted OR [95% CI] | P-value |
| Particulate matter (PM_10_) | 767 | 1.90 [0.99, 3.63] | 0.053 | 1.86 [0.97, 3.58] | 0.061 |
| Carbon monoxide (CO) | 716 | 2.10 [0.93, 4.73] | 0.074 | 2.24 [0.98, 5.12] | 0.054 |
| Benzene | 738 | 1.61 [0.84, 3.08] | 0.154 | 1.64 [0.85, 3.16] | 0.140 |
| Toluene | 738 | 1.15 [0.40, 3.35] | 0.793 | 1.14 [0.39, 3.31] | 0.812 |
| Maternal urine cotinine (vs non-smoker, n=249)  Passive smoker/exposed  Active smoker | 491  353 | 0.62 [0.33, 1.16]  1.04 [0.57, 1.91] | 0.135  0.892 | 0.62 [0.33, 1.18]  1.05 [0.55, 1.97] | 0.144  0.889 |
| β: β coefficient from linear regression model; OR: odds ratio; 95% CI: 95% confidence interval. Adjusted models: adjusted for maternal education, household income, maternal age at enrolment and maternal HIV infection. South African National Ambient Air Quality Standards based on an averaging period of 1 year: PM_10_: 40ug/m^3^, benzene: 5ug/m^3^, toluene: 240ug/m^3^; and based on a 1 hour average for CO: >30mg/m^3^ (not more than 88 hours). Maternal urine cotinine as a proxy for environmental tobacco smoke: <10 ng/ml (non-smoker), 10-499 ng/ml (passive smoker/exposed), or ≥500 ng/ml (active smoker). | | | | | |

| **Supplementary Table S2** Sensitivity analyses examining associations between indoor air pollution above ambient standards and environmental tobacco smoke (maternal urine cotinine) on infant birth outcomes, stratified by study site | | | | | | | | | |
| --- | --- | --- | --- | --- | --- | --- | --- | --- | --- |
| ***(A) Weight-for-age z-score at birth, among 1138 babies with birthweight data*** | | | | | | | | | |
|  | **Adjusted models, total sample** | | | **Adjusted models, restricted to Mbekweni** | | | **Adjusted models, restricted to Newman** | | |
|  | n | Adjusted β [95% CI] | P-value | n | Adjusted β [95% CI] | P-value | n | Adjusted β [95% CI] | P-value |
| Particulate matter (PM_10_) | 766 | -0.06 [-0.20, 0.08] | 0.414 | 403 | 0.01 [-0.19, 0.20] | 0.938 | 363 | -0.11 [-0.32, 0.09] | 0.282 |
| Carbon monoxide (CO) | 716 | -0.08 [-0.31, 0.15] | 0.491 | 410 | -0.11 [-0.44, 0.22] | 0.501 | 306 | 0.00 [-0.32, 0.32] | 0.994 |
| Benzene | 737 | -0.03 [-0.17, 0.12] | 0.715 | 393 | -0.09 [-0.29, 0.11] | 0.370 | 344 | -0.01 [-0.22, 0.20] | 0.929 |
| Toluene | 737 | -0.13 [-0.38, 0.12] | 0.323 | 393 | -0.29 [-0.63, 0.05] | 0.095 | 344 | 0.02 [-0.34, 0.39] | 0.896 |
| Maternal urine cotinine (vs non-smoker)  Passive smoker/exposed  Active smoker | 490  353 | -0.10 [-0.26, 0.06]  -0.54 [-0.71, -0.37] | 0.204  <0.001 | 312  91 | -0.11 [-0.29, 0.08]  -0.57 [-0.84, -0.31] | <0.001 | 178  262 | -0.05 [-0.36, 0.27]  -0.35 [-0.67, -0.04] | 0.772  0.025 |
| ***(B) Preterm versus full-term birth, among 1143 babies*** | | | | | | | | | |
|  | **Adjusted models, total sample** | | | **Adjusted models, restricted to Mbekweni** | | | **Adjusted models, restricted to Newman** | | |
|  | n | Adjusted OR [95% CI] | P-value | n | Adjusted OR [95% CI] | P-value | n | Adjusted OR [95% CI] | P-value |
| Particulate matter (PM_10_) | 767 | 1.05 [0.68, 1.61] | 0.836 | 404 | 0.84 [0.44, 1.59] | 0.589 | 363 | 1.18 [0.65, 2.11] | 0.589 |
| Carbon monoxide (CO) | 716 | 1.41 [0.74, 2.69] | 0.304 | 410 | 0.78 [0.26, 2.32] | 0.651 | 306 | 2.19 [0.94, 5.10] | 0.069 |
| Benzene | 738 | 1.14 [0.72, 1.79] | 0.578 | 394 | 0.95 [0.50, 1.80] | 0.865 | 344 | 1.34 [0.70, 2.57] | 0.371 |
| Toluene | 738 | 1.02 [0.47, 2.22] | 0.957 | 394 | 0.73 [0.21, 2.49] | 0.611 | 344 | 1.41 [0.50, 3.94] | 0.513 |
| Maternal urine cotinine (vs non-smoker)  Passive smoker/exposed  Active smoker | 491  353 | 1.03 [0.66, 1.60]  1.20 [0.75, 1.90] | 0.905  0.444 | 313  91 | 1.20 [0.71, 2.04]  1.20 [0.59, 2.44] | 0.497  0.620 | 178  262 | 0.59 [0.26, 1.35]  0.90 [0.41, 1.97] | 0.214  0.785 |
| ***(C) Respiratory distress versus no respiratory distress at birth, among 1143 babies*** | | | | | | | | | |
|  | **Adjusted models, total sample** | | | **Adjusted models, restricted to Mbekweni** | | | **Adjusted models, restricted to Newman** | | |
|  | n | Adjusted OR [95% CI] | P-value | n | Adjusted OR [95% CI] | P-value | n | Adjusted OR [95% CI] | P-value |
| Particulate matter (PM_10_) | 767 | 1.88 [0.98, 3.61] | 0.058 | 404 | 2.37 [0.95, 5.91] | 0.065 | 363 | 1.53 [0.60, 3.93] | 0.373 |
| Carbon monoxide (CO) | 716 | 2.19 [0.97, 4.98] | 0.061 | 410 | 2.18 [0.70, 6.81] | 0.182 | 306 | 2.44 [0.73, 8.17] | 0.149 |
| Benzene | 738 | 1.63 [0.85, 3.14] | 0.143 | 394 | 2.71 [1.04, 7.04] | 0.040 | 344 | 1.06 [0.40, 2.75] | 0.913 |
| Toluene | 738 | 1.14 [0.39, 3.31] | 0.811 | 394 | 1.72 [0.48, 6.19] | 0.408 | 344 | 0.60 [0.08, 4.73] | 0.628 |
| Maternal urine cotinine (vs non-smoker)  Passive smoker/exposed  Active smoker | 491  353 | 0.63 [0.34, 1.19]  1.04 [0.55, 1.95] | 0.155  0.904 | 313  91 | 0.62 [0.29, 1.29]  1.09 [0.43, 2.78] | 0.198  0.859 | 178  262 | 0.77 [0.20, 3.05]  1.31 [0.36, 4.73] | 0.711  0.685 |
| β: β coefficient from linear regression model; OR: odds ratio; 95% CI: 95% confidence interval. Adjusted models: adjusted for maternal education and household income. South African National Ambient Air Quality Standards based on an averaging period of 1 year: PM_10_: 40ug/m^3^, benzene: 5ug/m^3^, toluene: 240ug/m^3^; and based on a 1 hour average for CO: >30mg/m^3^ (not more than 88 hours). Maternal urine cotinine as a proxy for environmental tobacco smoke: <10 ng/ml (non-smoker), 10-499 ng/ml (passive smoker/exposed), or ≥500 ng/ml (active smoker). | | | | | | | | | |

| **Supplementary Table S3** Sensitivity analyses examining associations between environmental tobacco smoke (maternal urine cotinine, using alternative cotinine classifications) and birth outcomes | | | | | |
| --- | --- | --- | --- | --- | --- |
| ***(A) Weight-for-age z-score at birth, among 1138 babies with birthweight data*** | | | | | |
|  | n | **Unadjusted model** | | **Adjusted model** | |
|  |  | Unadjusted β [95% CI] | P-value | Adjusted β [95% CI] | P-value |
| Maternal urine cotinine  Non-smoker (<10 ng/ml)  Passive smoker/exposed (10-499 ng/ml)  Active smoker (≥500 ng/ml) | 249  490  353 | Ref  -0.10 [-0.26, 0.05]  -0.55 [-0.72, -0.39] | 0.196  <0.001 | Ref  -0.10 [-0.26, 0.06]  -0.54 [-0.71, -0.37] | 0.204  <0.001 |
| Maternal urine cotinine  Non-smoker (<10 ng/ml)  Passive smoker/exposed or active smoker (≥10 ng/ml) | 249  843 | Ref  -0.29 [-0.44, -0.14] | <0.001 | Ref  -0.27 [-0.42, -0.12] | <0.001 |
| Maternal urine cotinine  Non-smoker or passive smoker/exposed (<500 ng/ml)  Active smoker (≥500 ng/ml) | 739  353 | Ref  -0.48 [-0.61, -0.35] | <0.001 | Ref  -0.47 [-0.60, -0.33] | <0.001 |
| ***(B) Preterm versus full-term birth, among 1143 babies*** | | | | | |
|  | n | **Unadjusted model** | | **Adjusted model** | |
|  |  | Unadjusted OR [95% CI] | P-value | Adjusted OR [95% CI] | P-value |
| Maternal urine cotinine  Non-smoker (<10 ng/ml)  Passive smoker/exposed (10-499 ng/ml)  Active smoker (≥500 ng/ml) | 249  491  353 | Ref  1.09 [0.70, 1.68]  1.33 [0.85, 2.08] | 0.713  0.216 | Ref  1.03 [0.66, 1.60]  1.20 [0.75, 1.90] | 0.905  0.444 |
| Maternal urine cotinine  Non-smoker (<10 ng/ml)  Passive smoker/exposed or active smoker (≥10 ng/ml) | 249  844 | Ref  1.18 [0.79, 1.77] | 0.408 | Ref  1.09 [0.73, 1.65] | 0.665 |
| Maternal urine cotinine  Non-smoker or passive smoker/exposed (<500 ng/ml)  Active smoker (≥500 ng/ml) | 740  353 | Ref  1.26 [0.90, 1.77] | 0.186 | Ref  1.18 [0.83, 1.66] | 0.360 |
| ***(C) Respiratory distress versus no respiratory distress at birth, among 1143 babies*** | | | | | |
|  | n | **Unadjusted model** | | **Adjusted model** | |
|  |  | Unadjusted OR [95% CI] | P-value | Adjusted OR [95% CI] | P-value |
| Maternal urine cotinine  Non-smoker (<10 ng/ml)  Passive smoker/exposed (10-499 ng/ml)  Active smoker (≥500 ng/ml) | 249  491  353 | Ref  0.62 [0.33, 1.16]  1.04 [0.57, 1.91] | 0.135  0.892 | Ref  0.63 [0.34, 1.19]  1.04 [0.55, 1.95] | 0.155  0.904 |

| Maternal urine cotinine  Non-smoker (<10 ng/ml)  Passive smoker/exposed or active smoker (≥10 ng/ml) | 249  844 | Ref  0.79 [0.46, 1.37] | 0.409 | Ref  0.79 [0.45, 1.39] | 0.414 |
| --- | --- | --- | --- | --- | --- |
| Maternal urine cotinine  Non-smoker or passive smoker/exposed (<500 ng/ml)  Active smoker (≥500 ng/ml) | 740  353 | Ref  1.40 [0.85, 2.29] | 0.185 | Ref  1.39 [0.84, 2.31] | 0.200 |
| β: β coefficient from linear regression model; OR: odds ratio; 95% CI: 95% confidence interval. Adjusted models: adjusted for maternal education and household income. | | | | | |
